# Rice pest dataset supports the construction of smart farming systems

**DOI:** 10.1016/j.dib.2024.110046

**Published:** 2024-01-10

**Authors:** Luyl-Da Quach, Quoc Khang Nguyen, Quynh Anh Nguyen, Le Thi Thu Lan

**Affiliations:** FPT University, Can Tho campus, Cantho city, Vietnam

**Keywords:** Deep learning, Machine learning, Image segmentation, Computer vision, Rice disease

## Abstract

Rice holds a significant position in the global food supply chain, particularly in Asian, African, and Latin American countries. However, rice pests and diseases cause significant damage to the supply and growth of the rice cultivation industry. Therefore, this article provides a high-quality dataset that has been reviewed by agricultural experts. The dataset is well-suited to support the development of automation systems and smart farming practices. It plays a vital role in facilitating the automatic construction, detection, and classification of rice diseases. However, challenges arise due to the diversity of the dataset collected from various sources, varying in terms of disease types and sizes. This necessitates support for upgrading and enhancing the dataset through various operations in data processing, preprocessing, and statistical analysis. The dataset is provided completely free of charge and has been rigorously evaluated by agricultural experts, making it a reliable resource for system development, research, and communication needs.

Specifications Table

**Table:** 

Subject	Agricultural Sciences
Specific subject area	Image Processing, Image Identification, Image classification, object detection, computer vision, artificial intelligence, deep learning and reinforce learning
Data format	Raw Image
Type of data	Image
Data collection	Dataset collected image data from the Internet through datasets such as ImageNet [1], Microsoft COCO [2], images filtered from the IP102 dataset [3], and images gathered from the internet.
Data source location	All image data were labelled and evaluated by experts at the Cuu Long Delta Rice Research Institute, Vietnam.
Data accessibility	Repository name: Rice Pest Dataset Supports The Construction of Smart Farming Systems Data identification number: 10.5281/zenodo.8418217 Direct URL to data: https://zenodo.org/record/8418217 Instructions for accessing these data: Users can directly download data from the URL, then decompress the data set and can use it.
Related research article	Luyl-Da Quach, Khang Nguyen Quoc, Anh Nguyen Quynh and Hoang Tran Ngoc, “Evaluation of the Efficiency of the Optimization Algorithms for Transfer Learning on the Rice Leaf Disease Dataset” International Journal of Advanced Computer Science and Applications(IJACSA), 13(10), 2022. http://dx.doi.org/10.14569/IJACSA.2022.0131011

## 1 Value of the Data

• The dataset has a size of approximately 69.6 MB and comprises 3,156 images belonging to 10 classes. Each class contains various images related to rice pests and diseases, with the image resolution standardized to 312×312 pixel. This dataset is diverse as it was curated from various sources, resulting in differences in brightness, image resolution, and environmental conditions. This diversity poses challenges in processing for pest and disease detection, classification, and identification.
• This dataset, which classifies different types of rice pests and diseases, can be used to develop a system that supports the identification process, builds monitoring systems, and makes preliminary steps toward creating a smart farming system. The dataset contributes significantly to the development of systems for monitoring and classifying pests and diseases, such as surveillance and classification systems.
• The dataset has the potential to enhance the effectiveness of detecting and mitigating rice pests and diseases using smart agriculture systems. It also serves as a case study for developing eXplainable Artificial Intelligence algorithms, as seen in the classification of physiological states in tomatoes [4].
• Although there are several widely published datasets on harmful rice pests, most of these datasets are highly diverse. However, these datasets often contain repeated images, unclear distinctions between different types of harmful pests, and a lack of official management by agricultural research institutes. Therefore, the contribution of this research lies in combining expert input, filtering out duplicate image data, and standardizing image normalization. This aids in supporting subsequent studies by contributing and processing data related to larvae, eggs, and adults.
• This dataset is expected to yield more positive outcomes through human collection and evaluation by experts in the field of agriculture.

## 2 Background

According to the International Rice Research Institute's report, rice pests and diseases can cause up to a 37% reduction in rice yield for farmers, with the actual impact on production ranging from 24% to 41% depending on the specific agricultural conditions. This highlights the significant global concern regarding the influence of pests and diseases on rice production.

However, statistical data from scholarly sources indicates that from 2019 to the present, there have been approximately 17,000 research studies related to the keyword "rice leaf disease detection" and about 17,400 studies related to "rice pest detection" (content related to pests and rice but not necessarily containing the complete search phrase). Some notable studies on rice diseases are [5,6]. This demonstrates the scientific community's keen interest in developing a dataset to meet research needs in this field.

## 3 Data Description

The collected data includes images of harmful rice pests, primarily those affecting the leaves. For this research, image data was curated from various publicly available datasets on different types of rice pests, with a significant portion sourced from the IP102 dataset. In total, 3,156 images were gathered, representing 10 different rice pest species, which are Asiatic rice borer (Chilo suppressalis), Brown planthopper, Paddy stem maggot (Hydrellia sasakii), Rice gall midge (Orseolia oryzae), rice leafroller (Cnaphalocrocis medinalis), Rice leaf caterpillar, Rice leafhopper, Rice water weevil, Small brown planthopper, and yellow rice borer. These data were organized into ten folders, each in a zip file format, and the image format used was JPEG with a consistent size of 312 × 312 pixels.

**Fig. 1:**
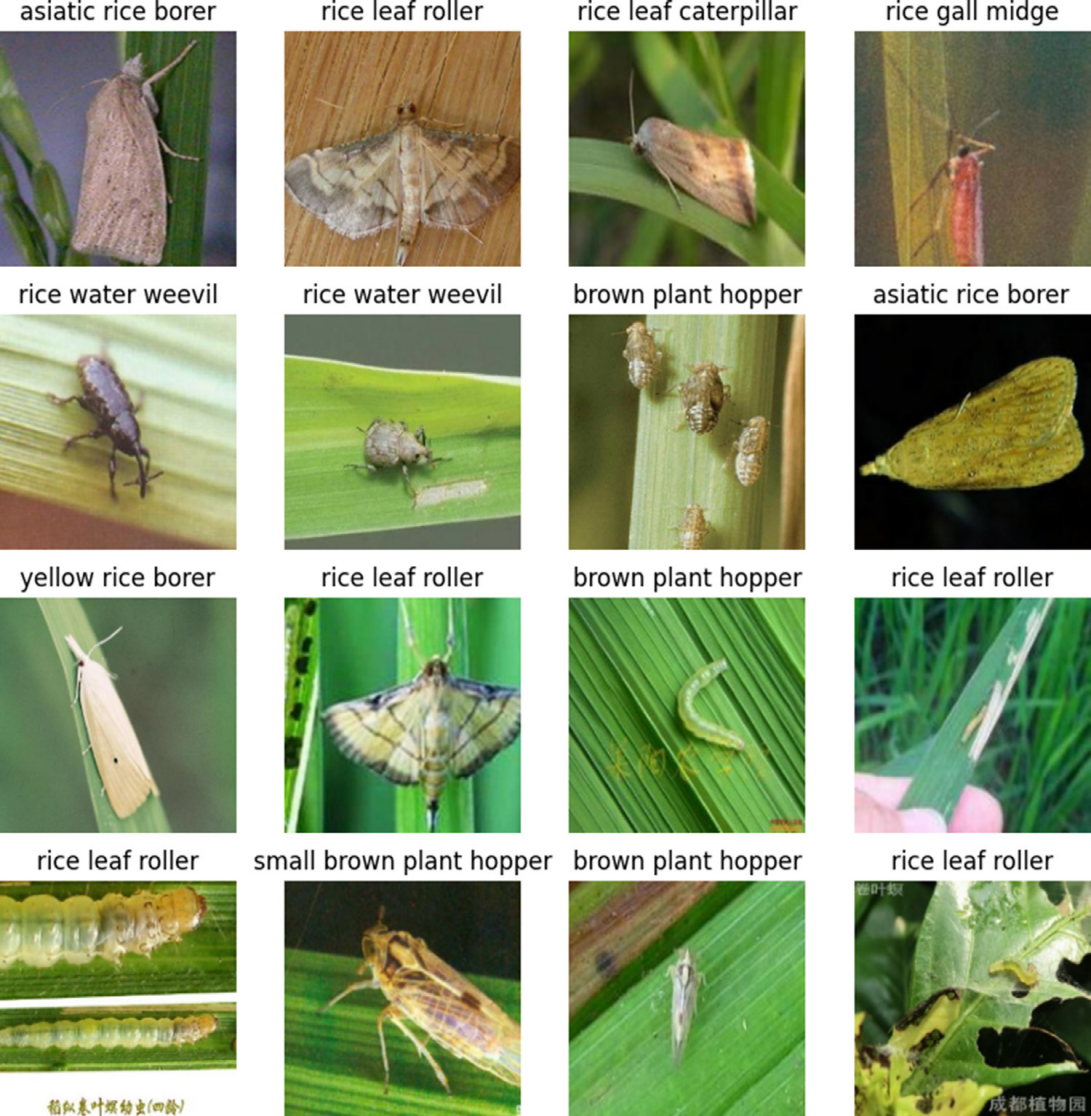
Image data sample in dataset.

To make data uploading and downloading simple, images were divided into separate folders by zip file. Fig. 1 illustrates sample data with 10 types of pests collected in the dataset.

## 4 Experimental Design, Materials and Methods

### 4.1 Field data collection

The data was collected and extracted from the IP102 dataset containing images of rice pests by a research team affiliated with the College of Computer Science, Nankai University, Tianjin, China, using sources from the Internet, ImageNet, and the Microsoft COCO dataset. During the evaluation and selection of image data, the research team observed that there were many noisy and feature-repetitive images. Consequently, the research team proceeded to gather and reevaluate the dataset of rice pest images. Subsequently, they sought the expertise of specialists in rice diseases from the Cuu Long Delta Rice Research Institute, Vietnam, to assess the reliability of the dataset.

The complexity of the datasets arises from the fact that the selected datasets are relatively intricate, requiring a focus on data relevant to harmful insect pests in rice. For example:

– The IP102 dataset [1] comprises over 75,000 images belonging to 102 categories, classifying insects hierarchically and affecting agriculture in general.
– The Microsoft COCO dataset [2] contains more than 330,000 images (with over 200,000 labeled images) featuring 81 object categories and 91 stuff categories.
– The ImageNet dataset [7] includes over 14 million images with more than 21,000 indexed sets.
– The remaining data sources, consisting of researched and evaluated images, amount to over 10,000 images.

**Fig. 2:**
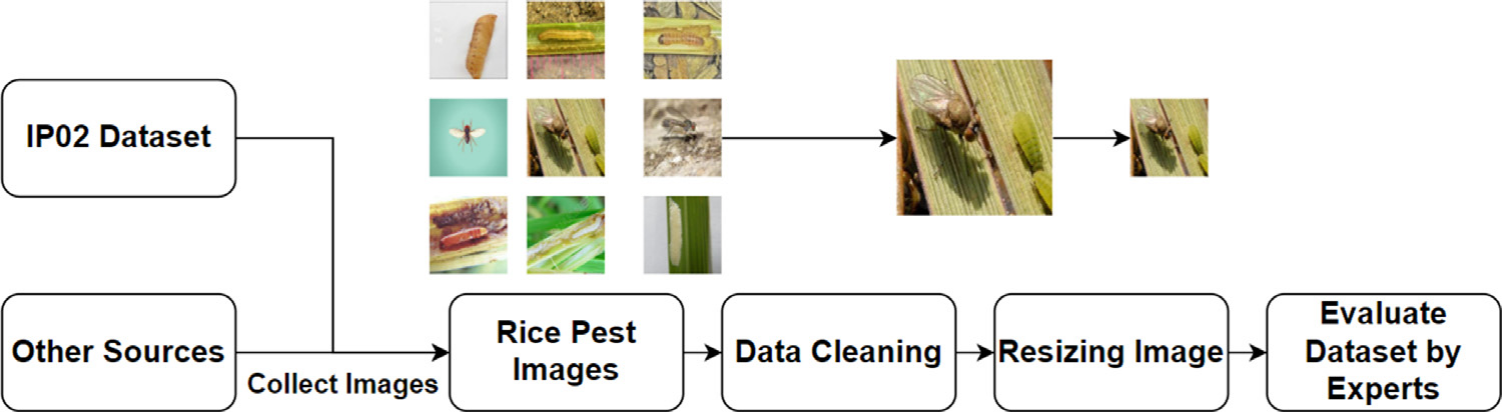
Illustration of the image collection and processing process for the pest dataset.

Therefore, the author's selection process from the extensive datasets is illustrated in Fig. 2. The results of the selection process are based on criteria outlined in Table 1.

**Table 1:** Statistics of original image data collected.

No.	Class (Common Name)	Scientific Name	Total Images	Adults	Eggs	Larvae
01	Asiatic rice borer	Chilo suppressalis	498	155	24	319
02	Brown planthopper	Nilaparvata lugens	346	335	7	4
03	Paddy stem maggot	Chlorops oryzae Matsumura	89	86	1	2
04	Rice gall midge	Orseolia oryzae Wood-Mason	217	217	0	0
05	Rice leaf roller	Cnaphalocrocis medinalis	153	108	1	44
06	Rice leaf caterpillar	Mythimna separata	716	108	1	44
07	Rice leaf hopper	Nephotettix spp.	244	243	1	0
08	Rice water weevil	Lissorhoptrus spp.	414	414	0	0
09	Small brown planthopper	Laodelphax striatellus	243	243	0	0
10	Yellow rice borer	Scirpophaga incertulas	236	213	18	5
Total			3.156	2.122	53	418

### 4.2 Data preprocessing

The original image data was processed, involving data cleaning, resizing, and evaluation of reliability by agricultural experts, as illustrated in Fig.2. The expert evaluation process is carried out as follows: one person labels the data, and another person reviews the data; this part of the process is conducted independently. The data is considered valid when both individuals assign matching labels. In the case of label discrepancies, the two experts reevaluate the issue with the support of a system contributed by the author team, following a standardized procedure to assist in machine learning and deep learning processes.

**Fig. 3:**
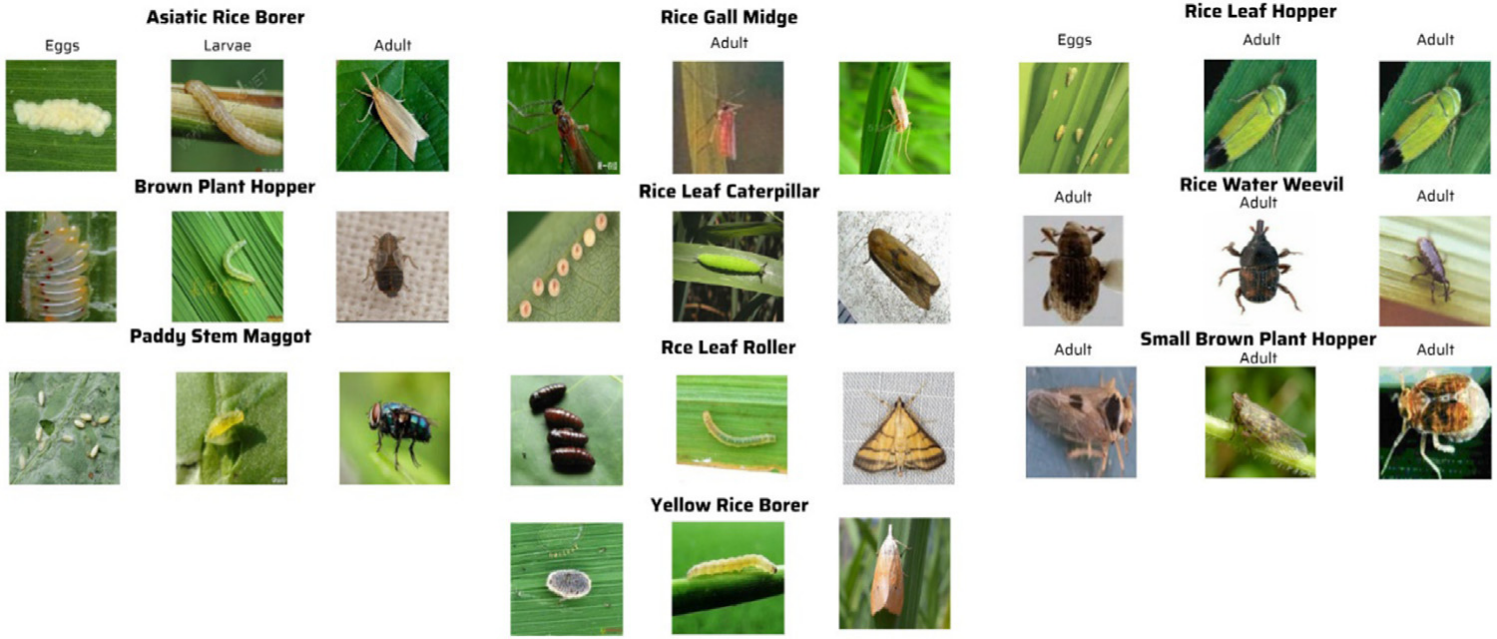
Illustrates the development process of each type of worm according to Adults, Eggs, and Larvae in the dataset.

An overview of the original data before processing is shown in Table 1. The images in the study have been re-checked for data duplication and have not undergone image transformations. The illustrative images depicting the classification of various types of insects in each developmental stage, such as larvae, eggs, and adults, are presented in Fig. 3.

## Limitations

Not applicable.

## Ethics Statement

This study did not conduct experiments involving humans and animals.

## CRediT authorship contribution statement

**Luyl-Da Quach:** Conceptualization, Methodology, Data curation, Writing – original draft, Visualization, Investigation, Writing – review & editing. **Quoc Khang Nguyen:** Conceptualization, Methodology, Data curation, Writing – original draft, Visualization, Investigation, Writing – review & editing. **Quynh Anh Nguyen:** Data curation, Visualization, Investigation. **Le Thi Thu Lan:** Conceptualization, Methodology, Data curation, Investigation, Writing – review & editing.

## Data Availability

Rice Pest Dataset Supports The Construction of Smart Farming Systems (Original data)
